# The effect of 5-aminoimidazole-4-carboxamide ribonucleoside (AICAR) on fatty acid oxidation in hepatocytes isolated from neonatal piglets

**DOI:** 10.1186/2049-1891-3-30

**Published:** 2012-10-17

**Authors:** Lin Xi, Gary Matsey, Jack Odle

**Affiliations:** 1 Laboratory of Developmental Nutrition, Department of Animal Science, North Carolina State University, Raleigh, NC 27695, USA; 2 Gordon Health Center 018, St Augustine’s College, 1315 Oakwood Avenue, Raleigh, USA

**Keywords:** Suckled neonatal pig, 5-aminoimidazole-4-carboxamide ribonucleoside (AICAR), Carnitine palmitoyltransferase (CPT), Acetyl-CoA carboxylase (ACC)

## Abstract

In the present study, the effect of 5-aminoimidazole-4-carboxamide ribonucleoside (AICAR) on long-chain fatty acid oxidation by hepatocytes isolated from suckled neonatal pig liver (a low ketogenic and lipogenic tissue) was tested. Incubation of hepatocytes with AICAR (0.5 mM) in the presence of 1 mM of carnitine and 10 mM of glucose for 1 hour at 37°C had no significant effect on total [1-^14^C]-palmitate (0.5 mM) oxidation (^14^CO_2_ and ^14^C-Acid soluble products (ASP)). Consistent with the fatty acid oxidation, carnitine palmitoyltransferase I activity and inhibition of its activity by malonyl-CoA (10 μM) assayed in cell homogenate also remained constant. However, addition of AICAR to the hepatocytes decreased ^14^CO_2_ production by 18% compared to control (p < 0.06). The reduction of labeled carboxylic carbon accumulated in CO_2_ caused a significant difference in distribution of oxidative products between ^14^CO_2_ and ^14^C-ASP (p < 0.03) compared with the control. It was also noticed that acetyl-CoA carboxylase (ACC) was increased by AICAR (p < 0.03), indicating that ACC might drive acetyl-CoA toward fatty acid synthesis pathway and induce an increase in distribution of fatty acid carbon to ^14^C-ASP. Addition of insulin to hepatocyte incubations with AICAR did not change the oxidative product distribution between CO_2_ and ASP, but further promoted ACC activity. The increased ACC activity was 70% higher than in the control group when citrate was absent in the reaction medium and was 30% higher when citrate was present in the medium. Our results suggest that AICAR may affect the distribution of metabolic products from fatty acid oxidation by changing ACC activity in hepatocyte isolated from suckled neonatal piglets; however, the basis for the increase in ACC activity elicited by AICAR is not apparent.

## Background

**Figure 1:**
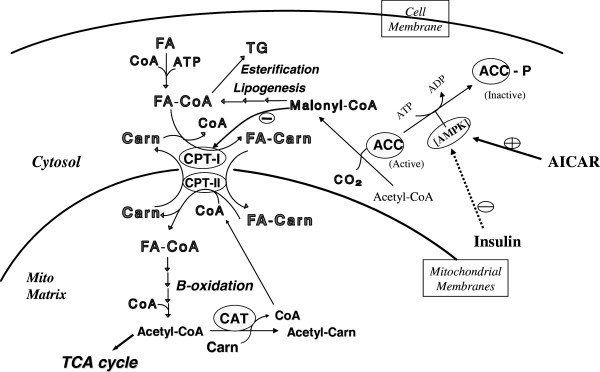
**Key roles of CPT I and ACC in fatty acid metabolism.** FA - Fatty acid, TG - Triglycerides, Carn – Carnitine, CPT - carnitine palmitoyltransferase, ACC - acetyl-CoA carboxylase, and AICAR - 5-aminoimidazole-4-carboxamide ribonucleoside.

The carnitine palmitoyltransferase (CPT) enzyme system is among the most important sites of regulation of hepatic long-chain fatty acid oxidation. Studies with fasted and diabetic animals have shown that the rise in fatty acid oxidation is mainly controlled by a decrease in malonyl-CoA concentration, a potent inhibitor of CPT I, and/or by a decrease in sensitivity of CPT I to the inhibition by malonyl-CoA (see Figure 1). A similar control mechanism also is found in neonatal rats and rabbits during the first 24 hours of life. In contrast, the role of CPT I in the regulation of fatty acid metabolism in neonatal piglets is not understood completely and displays interesting differences. The rate of long-chain fatty acid oxidation in liver mitochondria isolated from 24 h-old fasted pigs showed only a mild increase over newborns which was 70% lower than that observed in fasted adult rats. However, CPT I activity in pig liver mitochondria doubled between birth and 24 hour of age, and liver malonyl-CoA levels were very low due to a low hepatic lipogenesis in neonatal piglets. Duée et al. [1] reported that CPT I in neonatal pig liver was 50 times more sensitive than that from fasted adult rats. Studies from our laboratory [2-4] showed that the rate of long-chain fatty acid oxidation in hepatocytes, liver homogenates and mitochondria from neonatal piglets could be altered by changes in activity of CPT. The changes in CPT I activity, however, was not attributable to an increase in CPT I gene expression, but rather to a large decrease in the sensitivity of CPT I to malonyl-CoA inhibition. Moreover, the increase in fatty acid oxidation caused by the decrease of CPT I sensitivity to malonyl-CoA inhibition after birth is associated with food intake, emphasizing the importance of food intake in the regulation of fatty acid oxidation during early development. Because the pig CPT I protein is identified to be a natural chimera of the more typical mammalian liver and muscle CPT I isotypes, containing the liver CPT I binding site for acyl-CoA and the muscle CPT I binding sites for carnitine and malonyl-CoA, we have been interested in the role of malonyl-CoA in the kinetic modulation of hepatic CPT I in the 24-h old fed piglets. 

Malonyl-CoA, as the physiological inhibitor of CPT I, is the product of acetyl-CoA carboxylase (ACC). The activity of ACC plays a very important role in regulating carbohydrate and fatty acid metabolism, specifically the α isoform in lipogenic tissues and the β isoform, with a mitochondrial leader sequence, in non-lipogenic tissues [5]. The activity of ACC is controlled via a cycle of phosphorylation-dephosphorylation. The mechanism of interconversion of ACC from an active dephosphorylated form into a less active phosphorylated form as well as the hormonal control of ACC has been studied extensively during the past years. It is currently accepted that in intact hepatocytes and in the liver *in vivo*, the phosphorylation of ACC is mainly performed by a protein kinase termed the 5-adenosine monophosphate-activated protein kinase (AMPK). Studies indicated that insulin activates ACC in the liver through a dephosphorylation mechanism involving inhibition of AMPK, while the stimulation of AMPK inhibits fatty acid and cholesterol synthesis (Figure 1). 5-aminoimidazole-4-carboxamide ribonucleoside (AICAR) has been reported to be a specific activator of AMPK in intact cells. The effects of AICAR on fatty acid metabolism were tested in intact heart, muscle and liver cells [6-8]. It also was observed that a two-fold stimulation of palmitate oxidation and CPT activity occurred in hepatocytes isolated from rats incubated with AICAR along with a significant decrease in ACC activity and malonyl-CoA levels [9]. A recent study indicated that the AICAR increases mRNA expression of peroxisome proliferator-activated receptor (PPARα) target genes and peroxisome proliferator-activated receptor-γ coactivator (PGC)-1 in cultured muscle cells and mouse skeletal muscle [10]. There is limited information regarding hepatic ACC in swine particularly in neonatal pigs. To investigate the role of ACC in the regulation of fatty acid oxidation via a change of malonyl-CoA concentration, in this study we examined the effect of AICAR on fatty acid oxidation by hepatocytes isolated from suckled neonatal pigs.

## Methods

### Animal and hepatocyte isolation

All procedures were approved by the Institutional Animal Care and Use Committee of North Carolina State University. A total five suckled neonatal piglets (32 h-old, 1400 ± 200 g) from five sows were obtained from NCSU research farm unit II and hepatocytes were isolated using a two stage collagenase perfusion technique as described previously [11]. The cell yields were counted as ~1.75 x 10^9^ per liver and viability (Trypan Blue exclusion) was about 95% using this method. Cell and cell homogenate proteins were determined using biuret method [12].

### Hepatocyte incubation

After isolation cells (approximately 75 mg protein /mL) were incubated in Krebs-Henseleit bicarbonate buffer containing 10 mM glucose, 1 mM carnitine, and 3% (w/v) defatted BSA with different supplementations (I. 0.5 mM AICAR [9], II. 10 mU/mL insulin, and III. 0.5 mM AICAR plus 10 mU/ml insulin) and without supplementation (IV. control). Incubation was performed in 125-mL flasks at 37°C for 20 minutes with constant shaking under an atmosphere of O_2_/CO_2_ (19:1). Fatty acid oxidation and enzyme assays were performed either in the hepatocytes or hepatocyte homogenates after incubation.

### Fatty acid oxidation

For determination of fatty acid oxidation, 2.5 mL of incubation cells from each treatment in triplicate were transferred into 25-mL flasks. Subsequently, the reaction was started by addition of 3 μmoles of [1-^14^C]-palmitate (0.15 µCi/µmol) bound to BSA (3%) in 0.5 mL of Krebs-Henseleit bicarbonate buffer. The reaction was continued as described above for 30 minutes and stopped by addition of 0.5 mL of HClO_4 _(30%, vol/vol). The accumulation of [1-^14^C] in CO_2_ and acid soluble products (ASP) was measured using the techniques as described by Odle et al. [11]. The total oxidation rate was calculated as sum of CO_2 _and ASP.

### **Enzyme assays**

Hepatocytes (10 mL) were transferred into centrifuge tubes after incubation (total 50 minutes) and centrifuged at 50 x g for 3 minutes. The resultant cell pellet was homogenized in a buffer containing mannitol (220 mM), sucrose (70 mM), HEPES (2 mM) and EDTA (0.1 mM) using a glass-homogenizer with 3 strokes. The homogenate was used for the enzyme analysis directly as follows:

Malonyl-CoA sensitive CPT activity was measured using the method of Bremer et al. [13] slightly modified by our laboratory [14]. The assay was conducted at 37°C in a buffer containing KCl (75 mM), mannitol (50 mM), HEPES (25 mM), EGTA (0.2 mM), potassium cyanide (2 mM) and 1% BSA with cell homogenate (6 mg protein), palmitoyl-CoA (80 μM) and carnitine (1 mM). The reaction was started by addition of ^3^H - carnitine (4.5 µCi/μmol) and terminated by addition of 6% HClO_4_. Radioactivity in pamitoyl-carnitine was extracted by butanol and counted in a liquid scintillation counter as described previously [15].

ACC activity was assayed following the method described by Thampy and Wakil [16]. The reaction buffer (pH 7.5) contained HEPES (50 mM), ATP (4 mM), dithiothreitol (2 mM), MgCl_2 _(15 mM) and BSA (0.75 mg/mL) with or without citrate (15 mM). The assay was conducted at 37°C, initiated by addition of labeled KHCO_3 _(12.5 mM), and terminated by addition of 50 uL of HCl (6 N). Samples (0.4 mL) were evaporated at 65°C under a nitrogen stream and re-suspended in 0.5 mL of deionized H_2_O. The radioactivity in the re-suspended sample was determined via liquid scintillation.

### Statistics

Data were analyzed using the GLM procedure of SAS according to a randomized complete block design [17]. Results were expressed as least-squares means and standard error. Difference of the least-squares means between treatments groups were determined using the Tukey test and considered significantly when P < 0.05.

### Chemicals

[1-^14^C]-palmitate, ^3^H-carnitine and KH^14^CO_3_ were purchased from American Radiolabeled Chemicals Inc. (St. Louis, MO). Collagenase was purchased from Life Technologies (Grand Island, NY). All of other chemicals were purchased from Sigma-Aldrich Inc. (St. Louis, MO).

## Results

**Figure 2:**
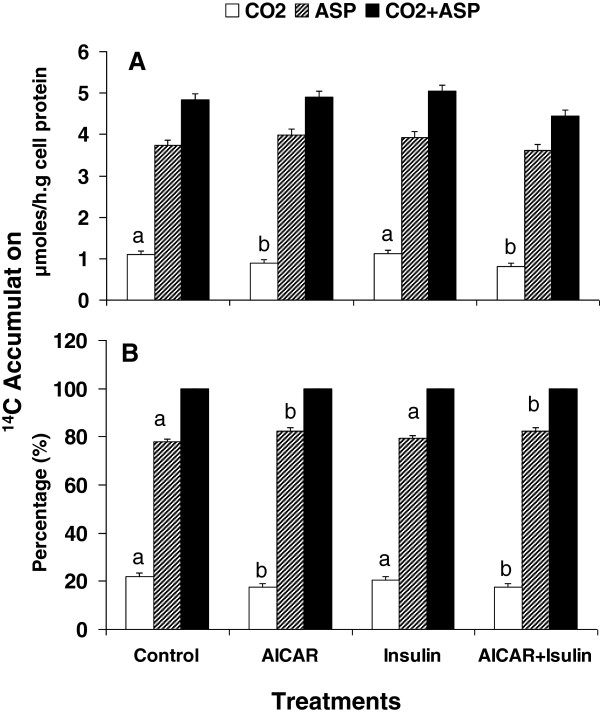
**Palmitate oxidation by hepatocyte isolated from suckled neonatal pigs. A**. ^14^C Accumulation in CO_2, _acid soluble products (ASP) and Total (CO_2_ + ASP). **B**. percentage of ^14^C accumulation in CO_2_ and ASP based on the total accumulation. Values presented as least-squares means ± SE. ^a,b^ Bars across the treatments with different letters differ (*P* < 0.05).

**Fatty-acid-oxidation:** Accumulation of ^14^C in CO_2_ was decreased by addition of AICAR to hepatocytes isolated from suckled neonatal pigs. ^14^CO_2_ production in cells incubated with AICAR or AICAR plus insulin was 18% lower than the control. There was no difference in CO_2_ production between control and addition of insulin only (Figure 2A). The accumulation of ^14^C in acid soluble products (ASP) remained relatively consistent for all of four treatments (*P* > 0.2; Figure 2A). No significant differences were observed in total palmitate oxidation (CO_2_+ASP) among the treatments (*P* > 0.08; Figure 2A). However, addition of AICAR to cells significantly affected the distribution of radioactivity accumulation between CO_2_ and ASP. The percentage of ^14^C-ASP was increased by 6% and the percentage of ^14^CO_2_ was decreased by 26% compared to control (*P* < 0.03; Figure 2B). Addition of insulin had no influence on the oxidative products distribution (*P* > 0.05).

**Figure 3:**
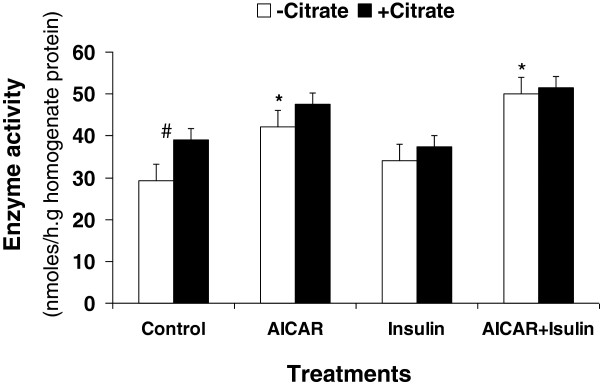
**Acetyl-CoA carboxylase activity in isolated hepatocyte homogenates from suckled neonatal pigs.** Values presented as least-squares means ± SE. * Significantly different from the control groups (*P* < 0.05). #Effect of citrate (*P* < 0.05).

**Figure 4:**
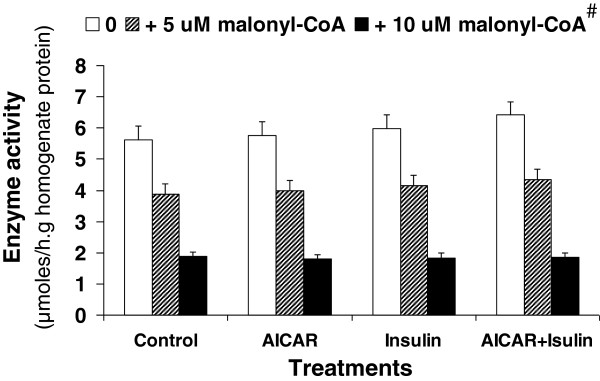
**Carnitine palmitoyltransferase activity in isolated hepatocyte homogenates from suckled neonatal pigs.** Values presented as least-squares means ± SE. * Significantly different between with and without malonyl-CoA (p < 0.05).

**Enzyme activity:** Acetyl-CoA carboxylase activity measured in hepatocyte homogenate was significantly affected by AICAR supplementation (Figure 3). The ACC activity was 45% higher in hepatocyte incubated with AICAR than in the control (*P* < 0.02). The enzyme activity was 70% higher in cells incubated with AICAR and insulin than in the control (*P* < 0.01). However, there was no difference between control cells and the cells treated with insulin only (*P* > 0.05). Addition of citrate to the cells increased the enzyme activity. The increase was higher in control cells than in the cells treated with AICAR, insulin or both. Carnitine palmitoyltransferase activity measured in the hepatocyte homogenates (Figure 4) showed no differences among the 4 treatments (*P* > 0.05). Addition of malonyl-CoA to cell homogenates significantly decreased the enzyme activity (*P* < 0.01). The decrease remained constant for all of the treatments.

## Discussion

Evidences from rodent studies have demonstrated that AICAR stimulates fatty acid uptake and fatty acid oxidation in muscle [10,18,19], heart [20] and liver [21]. The stimulation of fatty acid oxidation is recognized as the consequence of phosphorylating and inhibiting ACC, subsequently reducing the concentration of malonyl-CoA, the enzymatic product of ACC and the physiological inhibitor of CPT I. The reduction of malonyl-CoA concentration reduces CPT I inhibition, and thereby increases the fatty acid oxidation. Indeed, in adult animals it is well established that fatty acid oxidation is controlled mainly by the variation of malonyl-CoA concentration and the sensitivity of CPT I to malonyl-CoA inhibition in liver under many physiological conditions. In adult rat hepatocytes, reduction of malonyl-CoA concentration by glucagon significantly increases fatty acid oxidation. However, in this study the concentration of AICAR adapted from rodent species was sufficient to change malonyl-CoA concentrations in rat or mice, but there was no effect on the total [1-^14^C] palmitic acid oxidation in hepatocytes isolated from suckled neonatal piglets. In agreement with the fatty acid oxidation rate, the malonyl-CoA sensitive CPT activity and inhibition of its activity by malonyl-CoA assayed in cell homogenates remained unchanged among the treatments. The dampened responses of fatty acid oxidation to AICAR treatment could be associated with the species differences and the specific physiological status of the hepatocyte at the time of isolation. First, low lipogenesis and limited fatty acid oxidation capacity are observed in hepatocytes isolated from neonatal swine. Results from earlier studies demonstrated that the rate of lipogenesis is very low in isolated hepatocytes from both fed and fasted newborn pigs [22], suggesting that malonyl-CoA concentration could be negligible during early neonatal life. Meanwhile, the oleate oxidation and ketogenesis is about 70 and 80% lower in mitochondria isolated from newborn piglets than adult rats [1], and more than 90% of the oleate taken up by the hepatocyte converts to esterified fat [22], suggesting that newborn piglets have a low fatty acid oxidative capacity. However, the extremely low fatty acid oxidation is apparently not due to the CPT I inhibition, because the lipogenesis and malonyl-CoA concentration measured in hepatocytes isolated from newborn piglets is very low [1,22]. Therefore, the attenuated response to AICAR might be due to a low baseline concentration malonyl-CoA in the neonatal piglets hepatocytes. Secondly, evidence from literature indicates that the regulation of fatty acid oxidation during the neonatal period is different from adult animals. It is likely that the control of fatty acid oxidation is primarily effected by variation in sensitivity of CPT I to malonyl-CoA inhibition rather than by a change in malonyl-CoA concentration [23]. Indeed, we found that the considerable increase of fatty acid oxidation in hepatic mitochondria isolated from 24 h-old piglets was paralleled with a significantly decrease in sensitivity of CPT I to malonyl-CoA inhibition [4]. Moreover, the decrease in sensitivity of CPT I to malonyl-CoA inhibition was related to the food intake, because the IC_50_ obtained from 24 h-old fed piglets much higher than that from 24 h-old fasted and newborn piglets [4]. Similar results were also observed in our previous studied using hepatocytes and liver homogenate [2,24]. Because the hepatocytes isolated in this study were from 32 h-old fed piglets, the reduced response to AICAR might also be due an increased IC_50_ after the piglets suckled. Similar results were observed in muscle isolated from fasted rats [25], suggesting that the stimulation of fatty acid oxidation by AICAR depends on nutritional status. Thus, the stimulation of fatty acid oxidation by AICAR might be limited by the age-related physiological status.

Although AICAR did not change the total fatty acid oxidation, addition of AICAR to the cells decreased CO_2 _production by 18%, resulting in a significant difference in distribution of oxidative products between CO_2 _and ASP compared to the control. Consistent with the distribution change, we found that addition of AICAR increased ACC activity in hepatocytes, and the increase was promoted by adding insulin to the cells treated with AICAR. Inclusion of citrate in incubation medium also stimulated ACC activity in the cells, but the stimulation was higher in control cells than in cells treated with AICAR. These results suggest that the increased ACC activity induced by AICAR might drive the end product of beta-oxidation, acetyl-CoA, toward fatty acid synthesis, resulting in a decrease of CO_2 _production from fatty acid oxidation. As already discussed, the nutritional and physiological status of the isolated hepatocytes might be associated with the abrogated response of fatty acid oxidation to AICAR, but we have not evaluated the malonyl-CoA concentrations. If AICAR increases ACC activity, the malonyl-CoA concentration would be increased in the cells. It appeared that the increase of malonyl-CoA did not lead to a change in CPT I activity, the result might imply that the increases did not reach the inhibition level required by the CPT I in the cells with a high IC_50 _value due to the fed status. Even so, the phenomenon of increasing ACC activity could not be fully explained. Both isomers of ACCα and ACCβ are expressed in the liver, and ACCα sustains the regulation of fatty acid synthesis while ACCβ mainly controls fatty acid oxidation. The assay performed in this study could not distinguish the activity of ACCα and ACCβ, but their expression can be regulated by promoters at the transcriptional level in which nutritional status can play an important role. In addition to regulation at transcriptional level, ACCα and ACCβ are regulated by phosphorylation and dephosphorylation at the metabolic level. The phosphorylation is due to an increase of AMP levels when the energy status of the cells is low, resulting in the activation of AMPK. The cell energy level was high in this study, but AICAR is an activator of AMPK and its activation is considered to be independent of energy status of the cells [25]. Thus, the opposite influence of AICAR on ACC in newborn suckled pigs needs to be investigated further in both regulatory levels under the specific physiological and nutritional conditions. Particularly, the role of AMPK and insulin in regulation of ACC has not been studied and need to be examined in the neonatal pig. Further investigation is necessary for a better understanding of the energy and metabolic regulation mechanism in the newborn pigs. In summary, AICAR may affect the distribution of metabolic products from fatty acid oxidation in hepatocytes isolated from suckled neonatal pigs by changing ACC activity. The effect of AICAR on ACC activity will be impacted by citrate concentration in the cells.

## Abbreviations

ASP: Acid Soluble Products; AICAR: 5-Aminoimidazole-4-Carboxamide Ribonucleoside; ACC: Acetyl-CoA Carboxylase; CPT: Carnitine Palmitoyltransferase; AMPK: 5-Adenosine Monophosphate-activated Protein Kinase.

## Competing interests

The authors declare that they have no competing interests.

## Authors’ contributions

This study was conducted by GM and LX. The experimental design, the data statistical analysis and the manuscript was done by LX. JO participated in the experiment design, co-advised and coordinated the project summaries and publications. All authors read and approved the final manuscript.
